# Hepatic expression of sodium–glucose cotransporter 2 (SGLT2) in patients with chronic liver disease

**DOI:** 10.1007/s00795-022-00334-9

**Published:** 2022-09-21

**Authors:** Dan Nakano, Jun Akiba, Tsubasa Tsutsumi, Machiko Kawaguchi, Takafumi Yoshida, Hironori Koga, Takumi Kawaguchi

**Affiliations:** 1 Division of Gastroenterology, Department of Medicine, Kurume University School of Medicine, 67 Asahi-machi Kurume, Kurume, 830-0011 Japan; 2 Department of Pathology, Kurume University Hospital, Kurume, Japan; 3 Liver Cancer Division, Research Center for Innovative Cancer Therapy, Kurume University, Kurume, Japan

**Keywords:** Glucose transporter, SGLT2, Liver, Human tissue, Graphical model

## Abstract

Sodium–glucose cotransporter 2 (SGLT2) occurs in the proximal renal tubule cells. We investigate the hepatic expression of SGLT2 and its related factors in patients with chronic liver disease. This is a retrospective human study. The liver tissues were biopsied from patients with chronic liver disease (*n* = 30). The expression levels of SGLT2 were evaluated by immunostaining. Furthermore, the undirected graphical model was used to identify factors associated with hepatic expression levels of SGLT2. The SGLT2 expression was observed in not only the kidney, but also the liver in immunostaining (SGLT2 intensity: kidney 165.8 ± 15.6, liver 114.4 ± 49.0 arbitrary units, *P* < 0.01) and immunoblotting. There was no significant difference in hepatic expression of SGLT2 in the stratified analysis according to age, sex, BMI, and the severity of the liver disease. In the undirected graphical model, SGLT2 directly interacted with various factors such as sex, fatty change, neutrophil-to-lymphocyte ratio, triglyceride, hemoglobin A1c, creatinine, and albumin (partial correlation coefficient 0.4–0.6 for sex and 0.2–0.4 for others). The expression of SGLT2 was observed in the hepatocytes of patients with chronic liver disease. The undirected graphical model demonstrated the complex interaction of hepatic expression levels of SGLT2 with gender, inflammation, renal function, and lipid/glucose/protein metabolisms.

**Supplementary Information:**

The online version contains supplementary material available at 10.1007/s00795-022-00334-9.

## Introduction

Sodium/glucose cotransporter 2 (SGLT2) is a subtype of SGLT, which mediates glucose uptake across apical cell membranes [1]. SGLT2 occurs in the luminal membrane of proximal renal tubule cells and is responsible for more than 90% of glucose reabsorption from the glomerular ultrafiltrate in normoglycemia [1]. Renal protein expression of SGLT2 is increased by 40–80% in animal models of diabetes mellitus [2]. High messenger ribonucleic acid (mRNA) and protein expressions of SGLT2 are also observed in proximal renal tubule cells isolated from patients with diabetes mellitus than in cells from healthy individuals [3]. Thus, renal SGLT2 plays a crucial role in glucose metabolism.

SGLT2 is also known to play an important role in fatty acid metabolism [4]. The liver is the central organ involved in fatty acid metabolism. The liver stores fatty acids as triglycerides in hepatocytes and metabolizes fatty acids through beta-oxidation in the mitochondria of hepatocytes [5, 6]. The expression of SGLT2 has been observed in various human hepatoma cell lines [7, 8]. In hepatoma cells, SGLT2 uptakes glucose into cells. In addition, we previously reported that SGLT2 occurs in the mitochondria and regulates fatty acid metabolism in hepatoma cells [8]. However, the expression of SGLT2 and factors associated with hepatic SGLT2 expression remain unclear in humans. Extra-renal expression of SLGT2 has been investigated and its mRNA expression has been observed in various tissues including the heart and spleen in vitro and animal studies [9, 10]. In addition, protein expression of SGLT2 has been reported to occur in patients with traumatic brain injury [11]. These previous findings suggest the possible protein expression of SGLT2 in various tissues including the heart, spleen, and brain. However, the protein expression of SGLT2 in these tissues remains unclear in humans [8, 12–14].

This study aimed to investigate the expression of SGLT2 in patients with chronic liver disease. We also examined the extra-renal expression of SGLT2 in the human heart, spleen, and brain tissues in humans. We further investigated the direct factors associated with the hepatic expression level of SGLT2 in patients with chronic liver disease.

## Subjects and methods

### Study design and ethics

This is a retrospective study. The study protocol conformed to the ethical guidelines of the 1975 Declaration of Helsinki, as reflected by the prior approval of the Institutional Review Board of Kurume University School of Medicine (#21,104). An opt-out approach was used to obtain informed consent from the patients, and personal information was protected during data collection.

### Materials

The reagents were purchased from Cosmo Bio Co., Ltd. (Tokyo, Japan) unless otherwise indicated. Antibodies against SGLT2 were purchased from Proteintech Group, Inc. (24,654-1-AP, Rosemont, IL).

### Human tissues

The liver tissues were obtained from patients with chronic liver disease who underwent liver biopsy (*n* = 30). The kidney, heart, spleen, and brain tissues were obtained from necropsy patients with no impairment in each organ (each *n* = 5).

### Data collection

The variables were retrospectively reviewed using medical records. The following data were collected at the time of liver biopsy: age, sex, body mass index (BMI), red blood cell count, hemoglobin level, white blood cell count, neutrophil and lymphocyte ratio (NLR), platelet count, fasting blood glucose level, hemoglobin A1c (HbA1c) level, and serum levels of aspartate aminotransferase (AST), alanine aminotransferase (ALT), lactate dehydrogenase, gamma-glutamyl transpeptidase (GGT), alkaline phosphatase, cholinesterase, albumin, total bilirubin, prothrombin activity, total cholesterol, high-density lipoprotein (HDL)-cholesterol, triglyceride, blood urea nitrogen, and creatinine. We also calculated FIB-4 Index. In addition, the presence of hepatic steatosis and liver cirrhosis were evaluated by histological findings assessed by a board-certified pathologist (J.A.).

### Immunohistochemistry for SGLT2

Liver specimens were fixed in 10% formalin and embedded in paraffin. Consecutive 4-μm sections were stained with hematoxylin. Immunohistochemical staining was performed on paraffin-embedded tissues. Immunohistochemical staining was performed using SGLT2 (PGI 24,654-1-AP, Proteintech, Tokyo, Japan) and BOND-III (Leica Microsystems, Newcastle, UK). Proximal renal tubule cells of the kidney were used as a positive control for SGLT2.

### Image acquisition and processing

All samples were scanned at 40 × magnification using the Nano Zoomer (Hamamatsu Photonics K.K. Hamamatsu, Japan), which is a slide scanning system [15]. All images were saved in Tagged Image File Format files and processed using the FIJI software (version 2.0.0-rc-49/1.51d). Then, SGLT2 staining was separated from nuclear staining according to the color tone by using a color deconvolution plugin in FIJI [16]. This plugin separates the histological stains in the input image into different channels by applying the built-in H-3,3'-diaminobenzidine (DAB) vector to generate two images: color 1 (hematoxylin) and color 2 (DAB [SGLT2]). The SGLT2 channel was converted into an 8-bit grayscale image. Identical operations were applied to each image as follows: the background was subtracted by binarization. Binarization was performed using the default settings for the local thresholding algorithms (i.e. radius = 15 pixels, parameter 1 = 0, and parameter 2 = 0) [17].

### Measurement of SGLT2 staining intensity in the tissue

We measured the SGLT2 staining intensity by the oval region of interest. Three independent researchers measured the intensity thrice times in a tissue image with no clinical information (D.N., T.T., and T.K). The mean intensity of the nine measurements was used in the analysis.

### Classification of age and BMI

We classified all patients into the younger and older groups, or low and high BMI groups according to the median age (median age, 69.5 [59.0–79.3] years) and BMI (median 23.6, [20.9–24.9] years), respectively.

### Classification of the high and low SGLT2 groups

We classified all patients into the high and low SGLT2 groups according to the median hepatic expression level of SGLT2 (median SGLT2, 114 [73.2 -162.5]).

### Immunoblotting analysis

We examined the expression of SGLT2 by immunoblotting using kidney, normal liver, heart, spleen, and brain lysates. These lysates were purchased from Pro Sci inc (PSC 1706-02, 1718-01, 1301, 1306, and 1731-01, Poway, CA).

Immunoblotting was performed using the anti-SGLT2 antibody (sc-393350, Santa Cruz Biotechnology, Dallas, TX) as previously described [8].

### An undirected graphical model

An undirected graphical model was employed to explore the complex interaction between SGLT2 and the variables in graphs as previously described [18]. Two variables were connected by a line if the partial correlation coefficient was ≥0.2. An undirected graphical model was performed using JUSE-Stat Works/V5 (The Institute of Japanese Union of Scientists & Engineers, Tokyo, Japan).

### Statistics

Data are expressed as numbers or means ± standard errors. Differences between the two groups were analyzed using the Mann–Whitney *U* test. All statistical analyses were conducted by a biostatistician (M.K.).

## Results

### Patients’ characteristics

The patients’ characteristics are summarized in Table 1. The median age was 69.5 years and the male ratio was 66.7% (Table 1). The median BMI was 23.6 kg/m^2^. Hepatitis C virus was the major etiology of chronic liver disease followed by hepatitis B virus and non-virus. The prevalence of fatty liver and liver cirrhosis was 10.0% and 36.7% of all subjects, respectively (Table1).

**Table 1:** Patients’ characteristics

	Median (IQR)	Range (min–max)
Number	30	N/A
Age (years)	69.5 (59.0–79.3)	43–83
Sex (female/male)	33.3%/66.7% (10/20)	N/A
Body mass index (kg/m^2^)	23.6 (20.9–24.9)	16.9–30.9
Biochemical examinations		
Red blood cell (10^6^/μL)	431 (377–454)	319–501
Hemoglobin (g/dL)	13.6 (12–14.1)	9.6–15.2
White blood cell (/μL)	4550 (3675–5025)	2600–9700
Neutrophil (%)	55.7 (51.1–64.0)	37.6–77.5
Lymphocyte (%)	33.9 (26.0–39.2)	10.6–54.1
PT (%)	91.5 (87.8–99.8)	79–116
PT-INR	1.07 (1.01–1.09)	0.91–1.18
Platelet count (× 10^4^/µL)	13.9 (11.6–18.8)	4.4–31.2
AST (U/L)	33.0 (25.3–46.5)	20.0–86.0
ALT (U/L)	31.5 (22.5–46.3)	8.0–78.0
ALP (U/L)	263 (224–346.3)	143–739
GGT (U/L)	51.5 (27.8–121.3)	16–400
Cholinesterase (U/L)	235 (179–291)	56–342
Albumin (g/dL)	3.77 (3.38–4.12)	0.05–4.64
Total bilirubin (mg/dL)	0.76 (0.61–1.05)	0.42–2.2
HDL cholesterol (mg/dL)	49.5 (43.8–54.7)	23–75.6
Triglycerides (mg/dL)	92.5 (75.8–112)	44–449
Fasting glucose (mg/dL)	108.5 (93.5–130.5)	76.0–295
HbA1c (%)	5.7 (5.2–6.3)	4.7–7.8
BUN (mg/dL)	15.3 (12.3–17.5)	10.4–30.5
Creatine (mg/dL)	0.73 (0.64–0.85)	0.43–1.27
eGFR (mL/min)	74.6 (44.6–91.9)	14.2–156.4
FIB-4 index	3.0 (2.2–4.0)	1.0–16.4
FIB-4 index (< 2.67, 2.67 ≤)	37%/63% (11/19)	N/A
ALBI score	− 2.5(− 2.7 to − 2.1)	− 3.1–0.76
ALBI grade (1, 2, 3)	43.3%/46.6%/10% (13/14/3)	N/A
Pathological findings		
Liver cirrhosis (presence/absence)	36.7%/63.3% (11/19)	N/A
Fatty change (Fat: < 33%, 33% ≤ , 66% ≤ , 66% <)	90%/3.3%/3.3%/3.3% (27/1/1/1)	N/A

Data are expressed as median (interquartile range [IQR]), range, or number

*N/A* not applicable, *PT* prothrombin time, *PT-INR* prothrombin time International normalized ratio, *AST* aspartate aminotransferase, *ALT* alanine aminotransferase, *GGT* gamma-glutamyl transpeptidase, *HDL* cholesterol high-density lipoprotein cholesterol, *HbA1c* hemoglobin A1c; *FIB-4* fibrosis-4, *eGFR* estimated glomerular filtration rate

In the biochemical examination, the median serum levels of AST, ALT, alkaline phosphatase, and GGT were within reference values. However, the median level of albumin and platelet count was lower than the reference value and the median FIB-4 index was 3.0. The median levels of blood urea nitrogen, creatine, estimated glomerular filtration rate, HbA1c, HDL cholesterol, and triglyceride were within the reference values.

### Expression of SGLT2 on human tissues

**Fig. 1:**
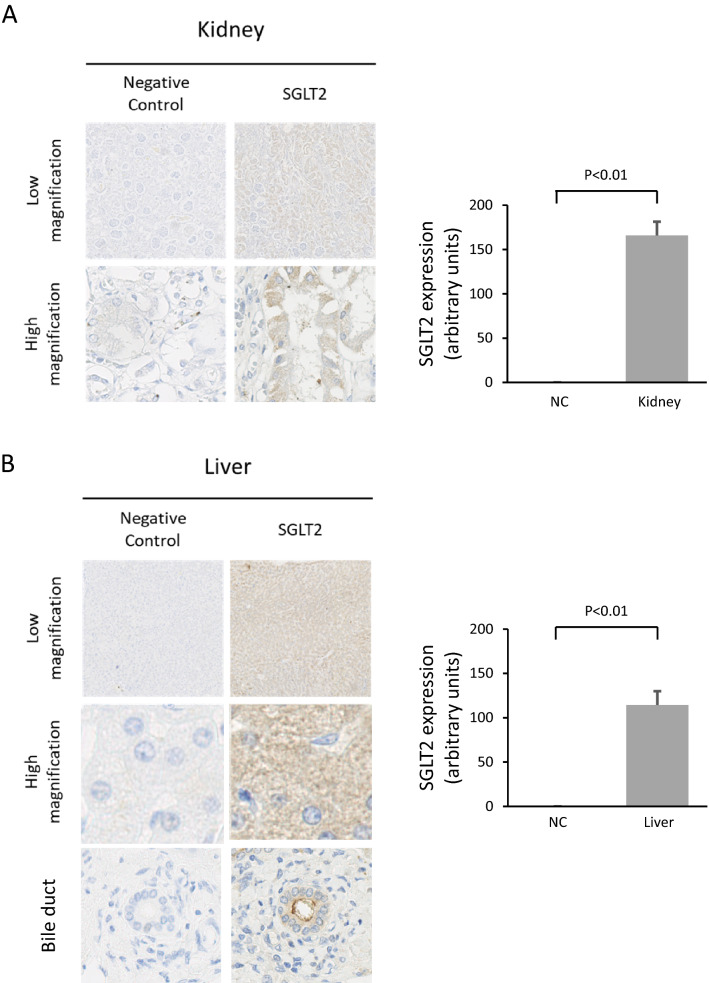

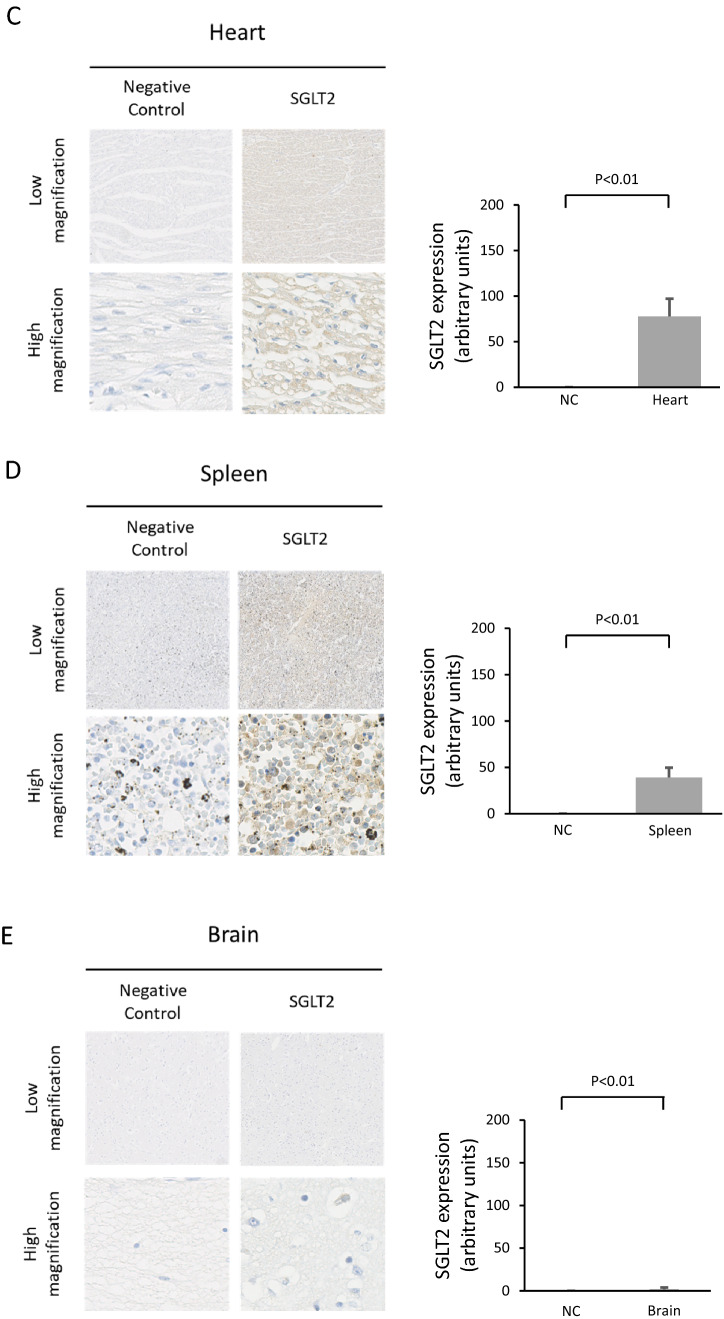
Expression of SGLT2 in human tissues evaluated by immunostaining. Blue indicates mitochondrial hematoxylin. Yellow indicates SGLT2. **A** Kidney (positive control), **B** liver, **C** heart, **D** spleen, **E** brain

In all tissues, a positive signal was not detected in the negative control examination, which lacks a primary antibody for SGLT2. Kidney tissue was used as the positive control. The expression of SGLT2 was observed in the proximal renal tubule cells and the expression level was significantly higher than that in the negative control (Fig. 1A).

In the liver, expression of SGLT2 was also observed. SGLT2 was found in the cytoplasm of hepatocytes. There was no lobular heterogeneity in SGLT2 expression. In addition, expression of SGLT2 was detected also at the apical membrane of bile duct cells (Fig. 1B). The expression levels in hepatocytes and bile duct cells were significantly higher than those in the negative controls (Fig. 1B).

In the heart tissue, expression of SGLT2 was found in the cytoplasm of the cardiomyocytes. In the spleen tissue, expression of SGLT2 also was found in the splenic cord. Weak expression of SGLT2 was observed in the brain tissue. The expression level of these tissues was significantly higher than the negative controls (Fig. 1C, D, E).

### Comparison of the expression level of SGLT2 in tissues

**Fig. 2:**
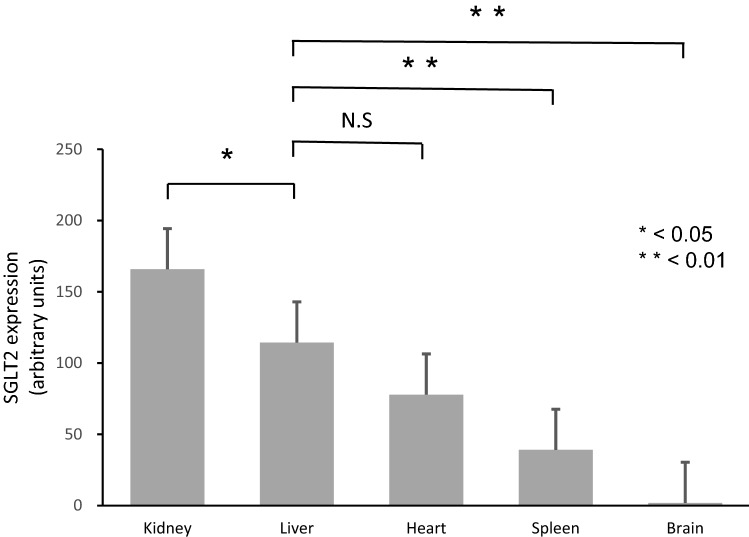
The difference in SGLT2 expression levels in the kidney, liver, heart, spleen, and brain tissues of humans

In immunostaining, the expression of SGLT2 differed with the tissues. Besides proximal renal tubule cells used as a positive control, a higher expression level of SGLT2 was seen in the liver. Lower and very low expression levels of SGLT2 were seen in the spleen and the brain (Fig. 2).

### Expression of SGLT2 in the lysate of human normal tissues

**Fig. 3:**
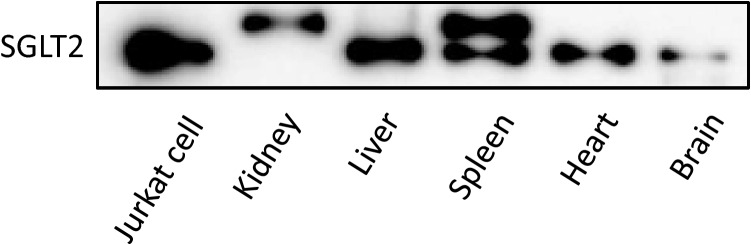
The expression of SGLT2 in the kidney, liver, heart, spleen, and brain was evaluated by immunoblotting. Jurkat cell is positive control of SGLT2 expression

In immunoblotting, expression of SGLT2 was observed in the kidney, liver, heart, spleen, and brain. Similar to the results of immunohistochemistry, a higher expression level of SGLT2 was seen in the liver1 (Fig. 3).

### The hepatic expression level of SGLT2 in the stratification analyses according to the severity of age, sex, BMI, and hepatic fibrosis

**Fig. 4:**
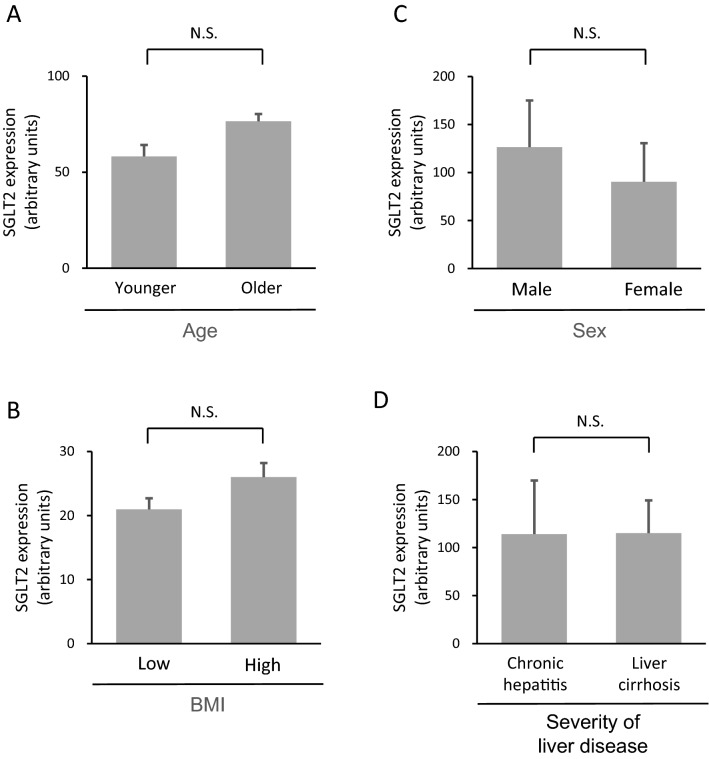
Differences in hepatic expression levels of SGLT2 according to **A** sex, **B** age, **C** BMI, and **D** severity of liver disease

We classified all patients into the younger and older groups, or low and high BMI groups according to the median age (median age, 69.5 [59.0 -79.3] years) and BMI (median 23.6, [20.9–24.9] years), respectively. There was no significant difference in hepatic expression of SGLT2 in the stratified analysis according to age, sex, and BMI (Fig. 4A, B, C). No significant difference was also observed in the expression levels of SGLT2 between patients with chronic hepatitis and liver cirrhosis (Fig. 4D).

### Associations between the hepatic expression level of SGLT2 and biochemical parameters

**Fig. 5:**
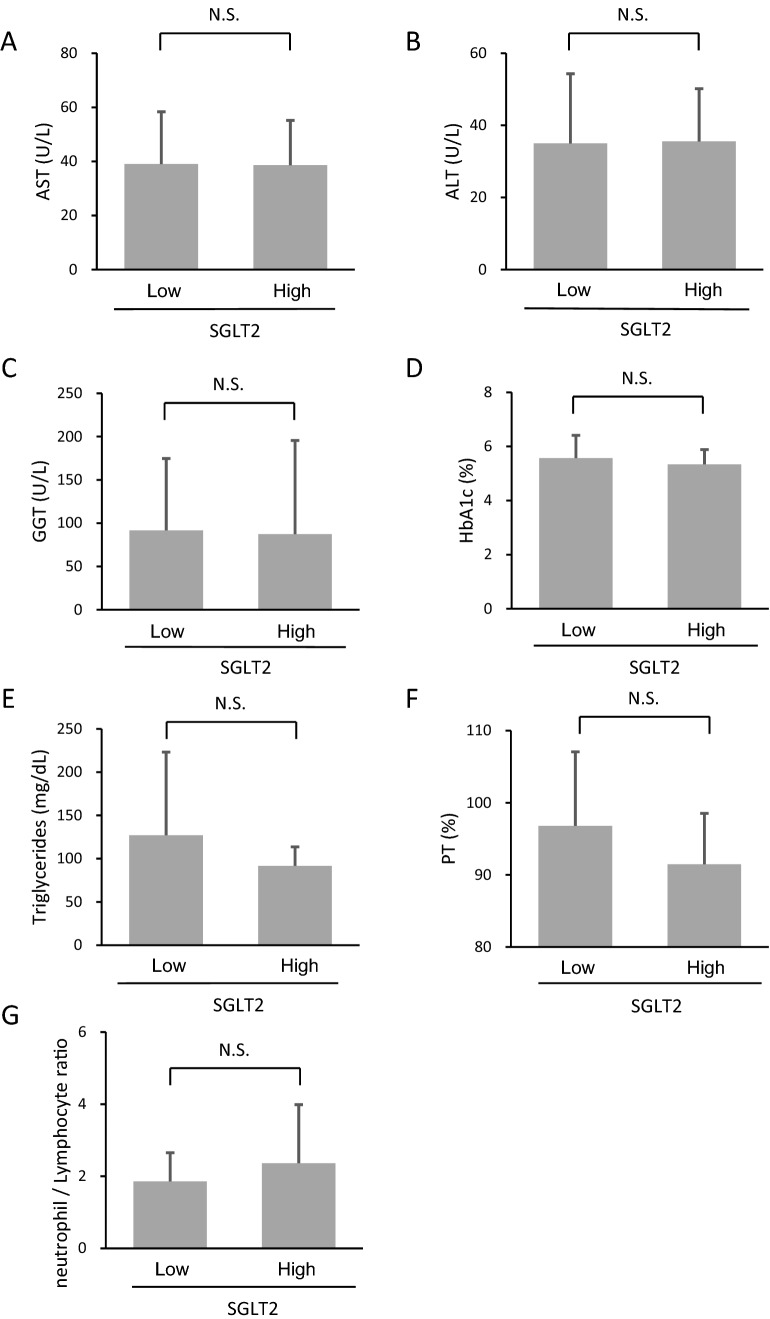
Differences in biochemical parameters between the high and Low SGLT2 groups. **A** AST, **B** ALT, **C** GGT, **D** HbA1c, **E** triglycerides, **F** prothrombin activity, and **G** NLR. *AST* Aspartate aminotransferase, *ALT* alanine aminotransferase, *GGT* gamma-glutamyl transferase, *HbA1c* hemoglobin A1c, *TG* triglyceride; NLR: Neutrophil /lymphocyte ratio

We classified all patients into high and low SGLT2 groups according to the median hepatic expression level of SGLT2. There was no significant difference in the serum levels of AST, ALT, and GGT between the High and Low SGLT2 groups (Figs. 5A, B, C). No significant differences were also observed in HbA1c, triglyceride, prothrombin activity, and NLR levels between the high and low SGLT2 groups (Figs. 5E, F, G).

### Factors associated with the expression of SGLT2 analyzed by an undirected graphical model

**Fig. 6:**
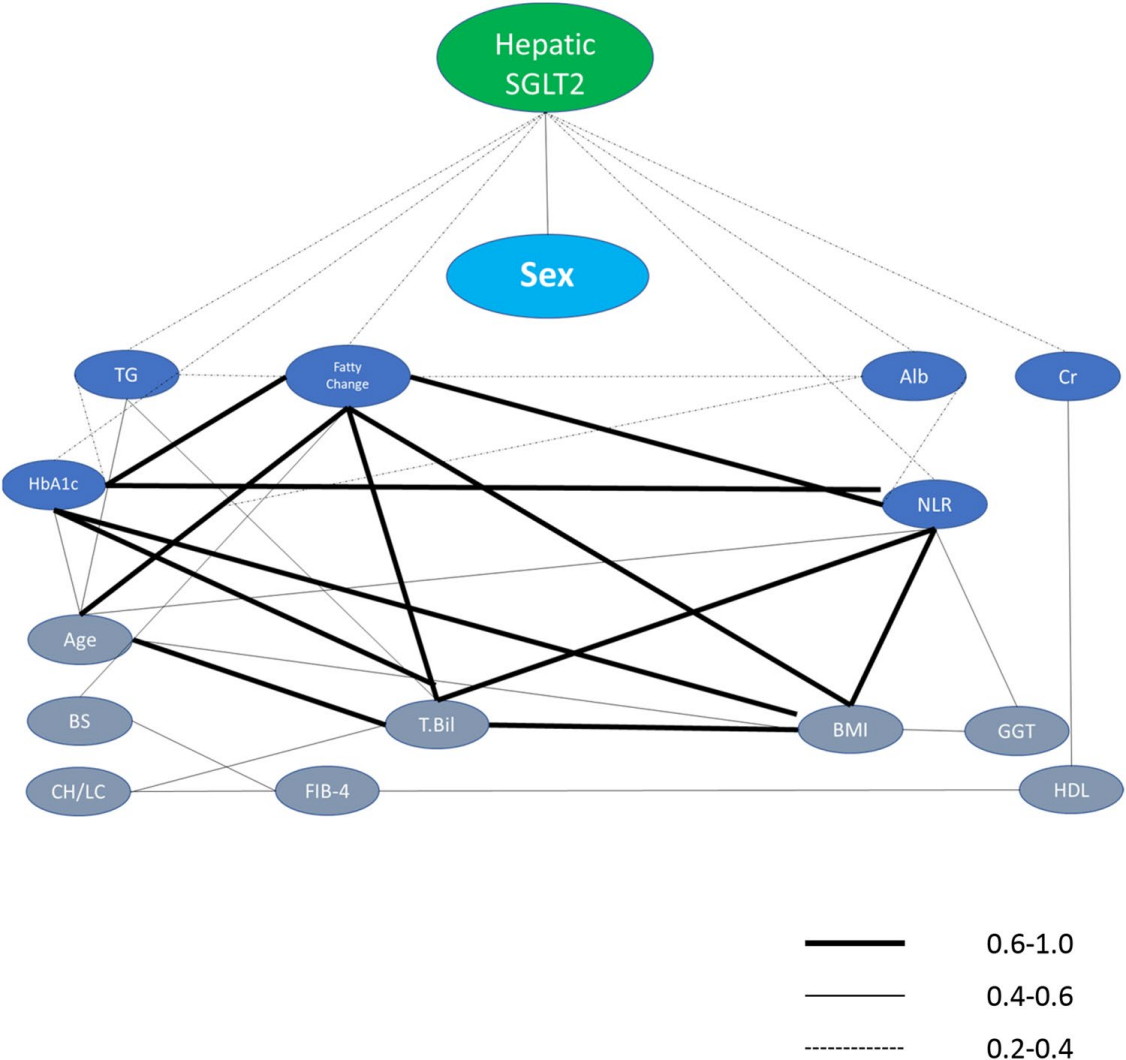
Undirected graphical model. The thick line, thin line, and dotted line indicate partial correlation coefficients of 0.6–1.0, 0.4–0.6, and 0.2–0.4, respectively. *SGLT2* sodium–glucose cotransporter 2; *Alb* albumin, *TG* triglyceride, *Cr* creatinine, *HbA1c* hemoglobin A1c, *T-Bil* total bilirubin, *BMI* body mass index, *GGT* gamma-glutamyl transferase, *BS* blood sugar, *CH/LC* chronic hepatitis/liver cirrhosis, *FIB-4* fibrosis-4 index, *HDL* high-density lipoprotein cholesterol

We employed an undirected graphical model to reveal the direct interaction between SGLT2 and various factors. In the undirected graphical model, SGLT2 directly interacted with seven factors including sex, fatty change, triglyceride, HbA1c, NLR, creatinine, and albumin. Of these factors, SGLT2 showed the strongest interaction with sex (Fig. 6).

## Discussion

In this study, we demonstrated that the expression of SGLT2 was observed in not only in the kidney, but also in the liver, heart, spleen, and brain. Of these organs, the hepatic expression of SGLT2 was the second -highest after the renal expression. In the undirected graphical model, SGLT2 directly interacted with various factors such as sex, fatty change, triglyceride, HbA1c, NLR, creatinine, and albumin.

The kidney was used as a positive control for immunostaining of SGLT2, and we reported that SGLT2 was found in the proximal renal tubule cells in this study. In rats, the SGLT2 gene is detected in the outer medulla of the kidney [19]. In addition, Rahmoune et al. reported the expression of SGLT2 in human proximal renal tubular cells isolated from the urine of patients with diabetes mellitus [3]. Moreover, Vrhovac et al. reported that the expression of SGLT2 is observed in the S1 and S2 segments of the human proximal tubule renal tissue [20]. Our results were in good agreement with previous reports. Thus, our immunostaining protocol is thought to be suitable to detect the protein expression of SGLT2 in human tissues.

In this study, we first demonstrated the expression of SGLT2 in the human liver. Expression of SGLT2 in the liver was second highest after that in the kidney. SGLT2 is thought to be found only in proximal renal tubule cells [19]. However, several recent studies have revealed the extra-renal expression of SGLT2. Bolla et al. reported that the expression of SGLT2 was observed in human duodenal mucosa in patients with diabetes mellitus [21]. Moreover, it is reported that various cancers have the expression of SGLT2 including pancreatic cancer, prostate cancer, and thyroid cancer [22, 23]. The expression of SGLT2 has also been observed in various human hepatoma cell lines [7, 8]. We also previously reported that SGLT2 is found in the mitochondria of Hep3B cells, regulating and regulating metabolic reprogramming and cell proliferation [8]. Furthermore, Zhao et al. reported that mRNA expression of SGLT2 was observed in the liver of cattle and the expression level was second highest after that in the kidney [9]. Moreover, Hasegawa et al. demonstrated that the accumulation level of luseogliflozin which binds to SGLT2 was second highest in the liver after that in the kidney in rats [24]. These previous studies support our findings that the expression of SGLT2 was found in human hepatocytes.

There was no significant difference in the hepatic expression of SGLT2 in terms of patient characteristics and biochemical parameters in our study. However, in the undirected graphical model, SGLT2 directly interacted with sex, fatty change, triglycerides, HbA1c, NLR, creatinine, and albumin. Of these factors, sex interacted the most with the expression level of SGLT2. An advantage of undirected graphical model analysis is bi-directional assessments and our results suggest the following two possibilities (1) sex was associated with SGLT2 expression or (2) SGLT2 expression was associated with sex. There was no significant difference in SGLT2 expression levels between men and women. This finding indicated that possibility 1 was denied and SGLT2 expression was associated with sex (possibility 2). To verify the possibility 2, we performed a sub-analysis and all subjects were classified into the lower or higher SGLT2 groups according to the cutoff value of 164.4, which was based on the receiver operating characteristic curve. In the higher SGLT2 group, the prevalence of males was significantly higher than in the lower SGLT2 groups (Supplementary figure).

Sabolic et al. reported that, in mice, the expression level of SGLT2 was male-dominant [25]. These sex differences may be related to testosterone levels. Sodium gradient-driven intravascular uptake of glucose was 2.5-fold higher in male mice than in female mice and testosterone treatment for females raised the levels to that in males [26]. Thus, these basic studies support our finding of an interaction between the hepatic expression of SGLT2 and sex in humans.

Moreover, Singh AK et al. performed a meta-analysis and reported the effect of SGLT2i was different in cardiovascular outcomes by gender [27, 28]. On the other hand, it is reported that SGLT2i adverse events including urinary tract/genital infection occur more frequently in women than men [29]. Thus, several previous studies reported gender differences in the effects of SGLT2 inhibitors.

We also reported that SGLT2 was found in the apical membrane of bile duct cells, cardiomyocytes, and splenic cells. Very low expression of SGLT2 was observed in the brain. We could not examine the detail of these expressions in this study. However, SGLT2 mRNA has been reported to be detected in the liver, cardiomyocytes, and spleen [9, 10]. Bile duct cells are known to reabsorb glucose from bile into the blood [30], suggesting the possible involvement of SGLT2. In addition, Ng et al. reported that the SGLT2 gene is observed in cardiomyocytes and is down-regulated by SGLT2 inhibitors, suggesting direct effects of SGLT2 inhibitors on cardiomyocytes [10]. Moreover, Hawley et al. reported that oral administration of SGLT2 inhibitor activates adenosine monophosphate-activated protein kinase in the spleen of mice [31]. Expression of SGLT2 has been reported to occur in patients with traumatic brain injury to increase glucose uptake increases in injured brain cells [11]. Thus, recent studies and our results suggest that SGLT2 may occur in various types of tissues.

This study has several limitations. First, this study was conducted in a single center with a small sample size. Second, all liver tissue samples were obtained from patients with chronic liver disease. Therefore, the expression of SGLT2 in normal human liver tissue remains unclear. Third, all samples were taken from Japanese individuals; therefore, racial differences in the expression of SGLT2 remain unclear. Accordingly, further study will be designed as an international multi-center study that includes a large number of samples with normal liver tissue specimens obtained by hepatic resection for metastatic liver tumors.

## Conclusion

In this study, we demonstrated that SGLT2 occurred not only in the kidney but also in the liver, heart, spleen, and brain. Expression of SGLT2 in the liver was second highest after that in the kidney. The undirected graphical model revealed the complex interaction of hepatic expression levels of SGLT2 with various factors such as gender, inflammation, renal function, and lipid/glucose/protein metabolisms.

## Supplementary Information

Below is the link to the electronic supplementary material.

Supplementary file1 (PPTX 3060 KB)
