# Supplementary figures and images for: Hepatic expression of sodium–glucose cotransporter 2 (SGLT2) in patients with chronic liver disease
Source: Med Mol Morphol. 2022 Sep 21;55(4):304–15. doi: 10.1007/s00795-022-00334-9 (PMC9606064; doi:10.1007/s00795-022-00334-9)

## Slide 1
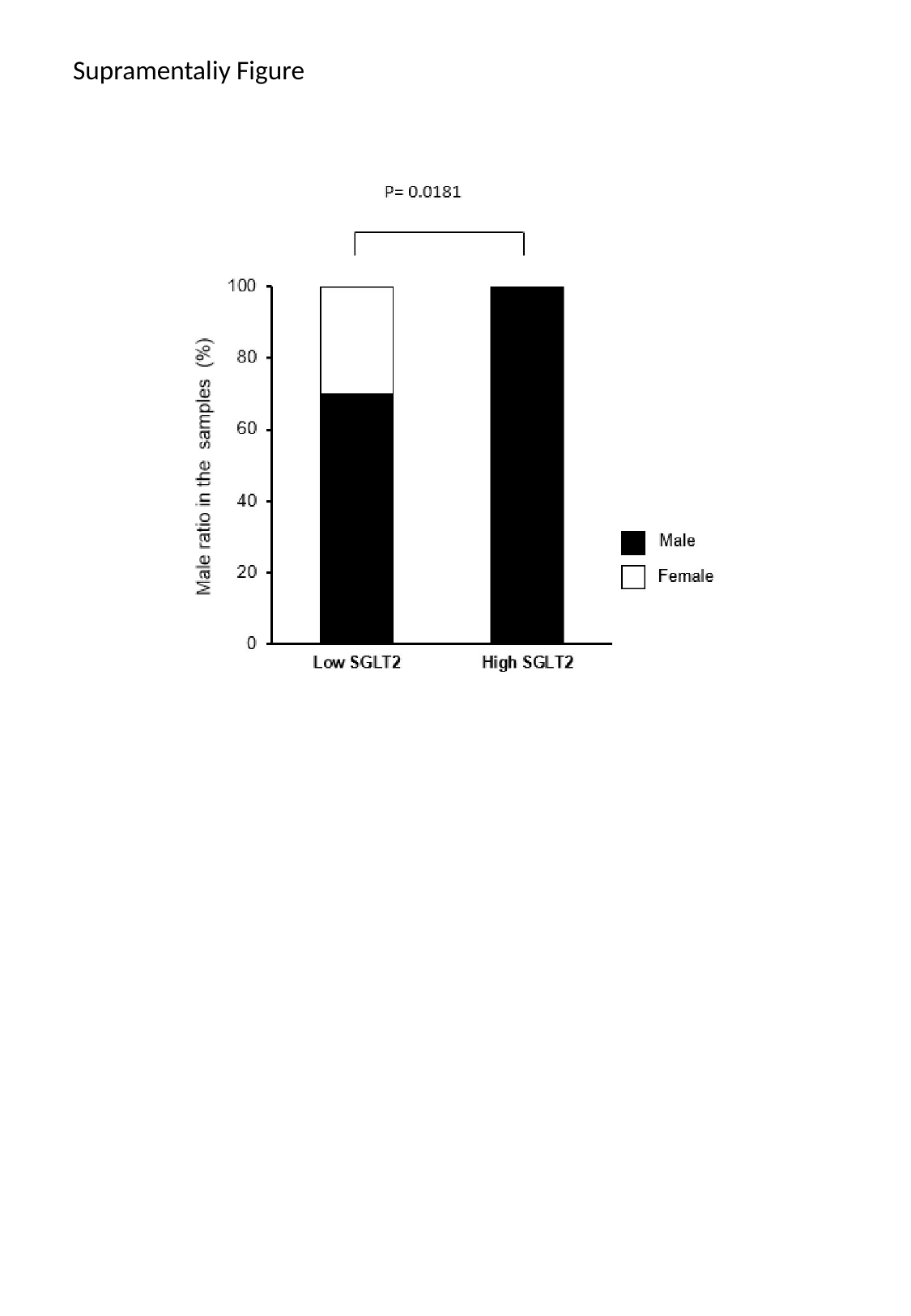

Supramentaliy Figure

Supplement: Supplementary file 1 — Supplementary file1 (PPTX 3060 KB) [file 795_2022_334_MOESM1_ESM.pptx]
